# Modulation of specificity protein 1 by mithramycin A as a novel therapeutic strategy for cervical cancer

**DOI:** 10.1038/srep07162

**Published:** 2014-11-24

**Authors:** Eun-Sun Choi, Jeong-Seok Nam, Ji-Youn Jung, Nam-Pyo Cho, Sung-Dae Cho

**Affiliations:** 1 Division of High-risk Pathogen Research, Korea Centers for Disease Control and Prevention, Osong, Republic of Korea; 2 Lee Gil Ya Cancer and Diabetes Institute, Gachon University, Graduate School of Medicine, Incheon 406-840, Korea; 3 Department of Companion and Laboratory Animal Science, Kongju National University, Yesan 314-701, Republic of Korea; 4 Department of Oral Pathology, School of Dentistry and Institute of Biodegradable Material, Institute of Oral Bioscience, Brain Korea 21 Project, Chonbuk National University, Jeon-ju 561-756, Republic of Korea

## Abstract

Cervical cancer is the third most common cancer and the third leading cause of death among women. However, the standard treatment for cervical cancer includes cisplatin, which can cause side effects such as hematological damage or renal toxicity. New innovations in cervical cancer treatment focus on developing more effective and better-tolerated therapies such as Sp1-targeting drugs. Previous studies suggested that mithramycin A (Mith) inhibits the growth of various cancers by decreasing Sp1 protein. However, how Sp1 protein is decreased by Mith is not clear. Few studies have investigated the regulation of Sp1 protein by proteasome-dependent degradation as a possible control mechanism for the regulation of Sp1 in cancer cells. Here, we show that Mith decreased Sp1 protein by inducing proteasome-dependent degradation, thereby suppressing cervical cancer growth through a DR5/caspase-8/Bid signaling pathway. We found that prolonged Mith treatment was well tolerated after systemic administration to mice carrying cervical cancer cells. Reduction of body weight was minimal, indicating that Mith was a good therapeutic candidate for treatment of cancers in which Sp1 is involved in promoting and developing disease.

Cancer initiation and progression are mediated through dysregulation of multiple signaling pathways. Therefore, the potential therapeutic effects achievable by targeting individual signaling pathways may be largely limited^1^. Targeting the divergence points of diverse signaling pathways may represent a promising therapeutic strategy for various cancers. Targeting transcription factors is particularly attractive because they are nodal points of multiple signaling pathways and are commonly deregulated in cancer^2^. Inhibition of excessive oncogenic transcription factor activity could be an effective strategy for new chemotherapeutic agents.

Specificity protein 1 (Sp1) is a zinc-finger transcription factor that regulates multiple cellular functions and promotes tumor progression by controlling expression of genes involved in cell cycle^3^, apoptosis^4^ and DNA damage^5^. Several studies demonstrated that Sp1 binds to GC-rich motifs of promoters and interacts with components of the general transcriptional machinery and co-activator complexes of multiple signaling pathways^6^. Increasing evidence suggests that aberrant expression or activity of Sp1 occurs in various cancers types^6^. Suppression of Sp1 levels reduces tumor growth in mice implanted with lung cancer cells^7^. Sp1 is directly involved in nicotine-induced lung cancer cell growth^8^. Therefore, it would be worthwhile to test promising cancer therapeutic drugs targeting Sp1 with less cytotoxic potency.

Mith, a selective Sp1 inhibitor, is a natural polycyclic aromatic polyketide isolated from Streptomyces strains^9^. Mith is used clinically as a chemotherapeutic agent to treat several cancer types including testicular carcinoma^10^ and chronic myeloid leukemia^11^. Mith inhibits binding of Sp1 to promoters, thereby inhibiting proto-oncogenes such as ha-RAS^12^ and c-Myc^13^; anti-apoptotic genes such as survivin^14^ and XIAP^15^; and pro-angiogenic genes such as VEGF^16^. However, regulation of Sp1 levels by proteasome-dependent degradation has not been investigated as a possible mechanism for controlling the amount of Sp1 in cancer cells. Here, we show that Mith decreased Sp1 protein levels by inducing proteasome-dependent degradation in cervical cancer cells.

Cervical cancer is a primary cancer of the uterine cervix and the second most common cancer diagnosed in women after breast cancer^17^. Although mortality rates have steadily decreased over the past decades because of early detection and screening, many patients have an unfavorable prognosis^18^. Cisplatin-based chemotherapy is gold standard treatment for metastatic cervical cancer^19^. However, cisplatin administration can cause gastrointestinal, hematological, or renal toxicity^20^. Cisplatin- induced toxicity often requires dose reduction, treatment delay, or discontinuation of therapy. Thus, finding less toxic and more effective targets and therapeutic drugs to treat cervical cancer is highly desirable.

In this study, we demonstrated that Mith significantly inhibited cervical cancer growth *in vitro* and *in vivo*. At the molecular level, Mith dramatically induced Sp1 degradation in a proteasome-dependent manner and suppressed growth of cervical cancer cells through a DR5/caspase-8/Bid signaling pathway. Taken together, our results support the hypothesis that suppression of cellular Sp1 levels by Mith is an effective therapeutic strategy for cervical cancer.

## Methods

### Antibodies and Reagents

Antibodies against cleaved caspase-3, 8, 9, PARP, Bax, Bak, Bad, Bim, Puma, Bcl-2, Bcl-xL, Mcl-1, DR5, DR4 and phospho-eIF4E were from Cell Signaling (Danvers, MA, USA) and Cox4 antibody was from Abcam (Cambridge, UK); cytochrome C antibody was from BD Biosciences (San Diego, CA, USA); MG-132 and antibodies for Sp1, α-tubulin and actin were from Santa Cruz Biotechnology, Inc. (Santa Cruz, CA, USA); DAPI, CHX and Mith was from Sigma-Aldrich Chemical Co. (St Louis, MO, USA); zVAD-fmk, a pancaspase inhibitor, was from R&D Systems (Minneapolis, MN, USA).

### Cell culture and Drug treatment

HEp-2 cells were from Kyungpook National University (Daegu, Korea) and KB cells were from American Type Culture Collection (Manassas, VA, USA). Cells were cultured in DMEM 100 U/mL each of penicillin and streptomycin and 10% FBS for HEp-2 cells and 5% FBS for KB in a humidified atmosphere containing 5% CO_2_ at 37°C. Equal numbers of cells were seeded and allowed to attach. At 50–60% confluence, cells were treated with DMSO or indicated concentrations of Mith diluted in DMEM with 5% FBS for HEp-2 cells and 2.5% for KB cells. Mith was dissolved in 0.1% DMSO.

### MTS assay

Cell viability was determined using CellTiter 96 Aqueous One Solution Cell Proliferation Assay Kits (Promega, Madison, WI, USA) according to the manufacturer's instructions. In brief, cells were seeded in 96-well plates and incubated with Mith. After treatment, 30 µl MTS solution was added to each well and cells were incubated for 2 h at 37°C. MTS solution was analyzed using a microplate reader (BioTeck Instruments Inc., Winooski, VT, USA) at 490 nm and 690 nm (background).

### Cell counting

Cells were seeded in 6-well plates at 2 × 10^5^ cells/well for HEp-2 cells and 5 × 10^5^ cells/well for KB cells. Cells were exposed to DMSO or Mith for 48 h, washed with 2 mL ice-cold phosphate buffered saline (PBS), dislodged with trypsin, and pelleted by centrifugation for 3 min at 800 rpm. Total attached cells were counted using a Neubauer's chamber (hemocytometer).

### Nuclear Staining with DAPI

HEp-2 and KB cells seeded in 60 mm^2^ dishes were treated with Mith for 48 h. Cells were harvested by trypsinization and fixed in 100% ethanol overnight at −20°C. Cells were washed three times in ice-cold PBS and fixed with 1 mL methanol solution at room temperature (RT) for 10 min. Fixed cells were stained with DAPI (2 μg/mL) solution and deposited on slides at RT in the dark. DAPI-stained cell morphology was observed under a fluorescence microscope.

### Preparation of cytosol and mitochondrial fraction

Cytosolic and mitochondrial fractions were isolated by digitonin or Triton X-100. HEp-2 and KB cells were washed with ice-cold PBS and cell pellets were resuspended for 1 min at RT in plasma membrane extraction buffer containing 0.05% digitonin. Following centrifugation at 13,000 rpm at 4°C for 5 min, supernatants containing cytosolic proteins were collected and mitochondrial pellets harvested by centrifugation at 13,000 rpm at 4°C for 3 min. Mitochondrial protein pellets were resuspended in plasma membrane extraction buffer containing 0.5% Triton X-100. Supernatants containing mitochondrial proteins were collected from the last centrifugation.

### Measurement of mitochondrial membrane potential

The mitochondrial membrane potential (ΔΨm) was determined using the mitochondria-specific lipophilic, cationic fluorescence compound JC-1 (Stratagene, La Jolla, CA, USA). HEp-2 and KB cells were seeded into 60 mm^2^ dishes. After treatment, cells were washed with PBS, dislodged with trypsin, and pelleted by centrifugation for 3 min at 800 rpm. Pellets were resuspendend in 500 μL 1X JC-1 staining reagent at 37°C for 30 min, followed by washing with ice-cold PBS. Cell-associated fluorescence was measured with fluorescence plate reader.

### Western blot analysis

Whole cell lysates were extracted with lysis buffer and protein concentrations were measured by the bicinchoninic acid (BCA) method using bovine serum albumin as standard. Samples containing equal amounts of protein were separated by SDS-PAGE and transferred to PVDF membranes (Bio-Rad Laboratories). The membranes were blocked with 5% skim milk in TBST at RT for 1 h. Membranes were incubated with primary antibodies overnight at 4°C and HRP-conjugated secondary antibodies for 90 min at RT. Antibody-bound proteins were detected using an ECL Western Blotting Luminol reagent (Santa Cruz Biotechnology Inc.). Bands were visualized by an LAS-500 imaging system.

### Reverse transcriptase polymerase chain reaction (RT-PCR)

Total RNA was extracted from Hep-2 and KB cells using easy-BLUE Total RNA Extraction Kits (iNtRON, Daejeon, Korea) following the manufacturer's recommendations. For reverse transcriptase polymerase chain reaction (RT-PCR), 2 μg total RNA was mixed with diethylpyrocarbonate (DEPC)-treated water for a total volume of 20 μL in Maxime PreMix Kit buffer. Reverse transcription was 60°C for 1 h and inactivation of RTase was 96°C for 5 min. Target cDNA was amplified using primers: sense 5′-ATG GGG GCA ATG GTA ATG GTG G-3′, antisense 5′-TCA GAA CTT GCT GGT TCT GTA AG-3′ for Sp1; and sense 5′-GTG GGG CGC CCC AGG CAC CA-3′, antisense 5′-CTC CTT AAT GTC ACG CAC GAT TTC-3′ for *β*-actin. Sp1 amplification was for 28 cycles (1 min at 94°C, 1 min at 58°C, and 1 min 30 sec at 72°C), and *β*-actin amplification was for 25 cycles (1 min at 94°C, 1 min at 60°C, and 1 min 30 sec at 72°C).

### Sp1 small interfering RNA

On TARGET plus SMART-pool small interfering RNA (siRNA) sequences targeting Sp1 and non-targeting control were purchased from Dharmacon Research (Lafayette, CO, USA). Transfection was according to the manufacturer's instructions. HEp-2 and KB cells were seeded at 50%–60% confluence in 6-well plates and transfected transiently with 25 or 50 nM siRNA using DharmaFECT2 transfection reagent (Thermo Scientific, Lafayette, CO, USA). After transfected for 6 or 48 h, HEp-2 and KB cells were analyzed for apoptotic effects using western blots and DAPI staining.

### Nude mouse xenograft

Female nude mice were from Orient Ltd. (Suwon, Korea). All animal studies were conducted according to protocols approved by the Institutional Animal Care and Use Committee of the university. KB cells were suspended in sterile PBS and injected subcutaneously into the right flank of mice. Mice were randomized into two groups containing five mice each and treated with 0.2 mg/kg/day of Mith (i.p.) three times per week for 29 days. Control mice received an equal volume of vehicle. After 29 days, bodies, organs and tumors were weighed and tumor volumes determined. Tumors were measured along the two diameter axis with calibers to allow calculation of tumor volume as V = π/6{(*D* + *d*)/2}^3^, where D and d are larger and smaller diameters, respectively.

### TUNEL assay

Tumor tissues were examined using a Dead-End Colorimetric TUNEL System (Promega) according to the manufacturer's instructions. After dewaxing and rehydration, tissue sections were treated with proteinase K for 15 min at RT. Endogenous peroxidase was blocked with 0.3% H_2_O_2_ in PBS for 5 min. Digoxigen dUTP end-labeled DNA was detected using an antidigoxigenin-peroxidase antibody, followed by peroxidase detection with 0.05% DAB containing 0.02% H_2_O_2_. Tissue sections were counterstained with methyl green. Brown apoptotic bodies in tumor sections of control and Mith-treated mouse samples were counted using a Nikon Eclipse E800 microscope (Nikon Inc. Melville, NY, USA).

### Histopathological examination

Tumors and liver, kidney, heart, lung and brain were fixed in 10% neutral buffered formalin, embedded, sectioned (5 μm) and stained with hematoxylin and eosin (H&E). Histopathological changes were observed using a Nikon Eclipse E800 microscope.

### Immunohistochemistry

Tissue sections were fixed with 10% formaldehyde, embedded in paraffin and cut into 4 mm sections. Sp1 staining used Envision kits (DAKO, Carpinteria, CA, USA). Sections were deparaffinized with xylene, dehydrated with ethanol and heated in 0.01 M citrate buffer (pH 6.0). Endogenous peroxidase was inactivated with 3% H_2_O_2_ for 10 min at RT and sections were blocked with 3% normal goat serum in 0.2 M PBS (pH 7.4). Samples were incubated with anti-Sp1 at RT for 1 h. Secondary antimouse antibody-coated polymer peroxidase complexes (DAKO) were applied for 30 min at RT, followed by treatment with substrate/chromogen (DAKO) and incubation for 5–10 min at RT. Slides were counterstained with hematoxylin.

### Statistical analysis

Quantitative data were presented as mean ± SD and were analyzed by Student's two-tailed *t-*test. A value of *p* < 0.05 was considered statistically significant.

## Results

### Mith dose-dependently inhibits growth and induces apoptosis in cervical cancer cells

**Figure 1:**
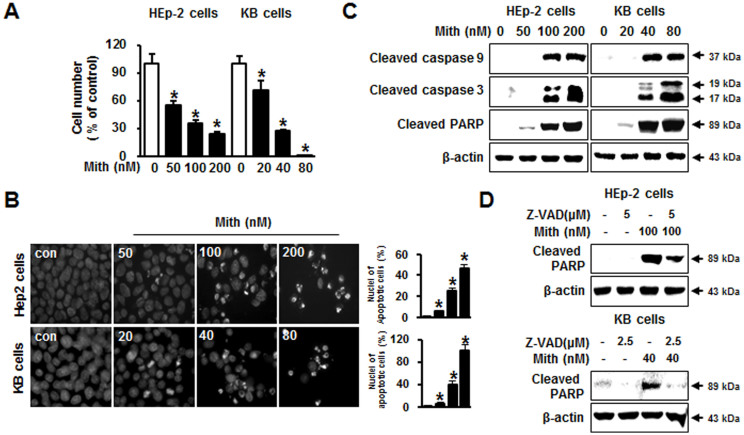
Effect of Mithramycin A (Mith) on cell viability and apoptosis in HEp-2 and KB cells. HEp-2 and KB cells were treated with or without Mith for 48 h. (A) Cell numbers were obtained by direct cell counting with a hemocytometer. (B) By fluorescence microscopy (magnification X400), HEp-2 and KB cells exhibited nuclear fragmentation and condensation after DAPI staining. (C) HEp-2 and KB cells were treated with Mith as indicated for 48 h before western blots of whole cell lysates detected caspase 3, poly(ADP-ribose) polymerase (PARP) and cleavage of caspase 9. (D) HEp-2 and KB cells were preincubated with pancaspase inhibitor zVAD-fmk for 1 h before Mith treatment and western blot to detect cleaved PARP. β-actin was the internal control. Results are mean ± SD from three independent experiments. * P < 0.05.

To assess the antiproliferative effects of Mith on cervical cancer cells, two cervical cancer cell lines with different genetic backgrounds were grown with or without treatment with Mith at different concentrations. Mith inhibited HEp-2 and KB cell growth in a concentration-dependent manner after 48 h (Fig. 1A). Apoptotic cell death was qualitatively estimated by DAPI staining for nuclear condensation and fragmentation. Mith led to significant DNA fragmentation compared to untreated controls (Fig. 1B). Western blots revealed that Mith treatment induced activation of initiator (caspase-9), effector caspases (caspase-3), and PARP (substrate for caspase-3) in both HEp-2 and KB cells (Fig. 1C). To determine if Mith-induced cell death was dependent on caspase activation, HEp-2 and KB cells were preincubated with the broad-spectrum caspase inhibitor zVAD-fmk before Mith exposure. Pretreatment of cells with zVAD-fmk attenuated Mith-induced PARP cleavage, suggesting that Mith induced caspase-dependent apoptosis in both HEp-2 and KB cells (Fig. 1D).

### Mith induces mitochondria-mediated apoptosis through DR5/caspase-8/Bid signaling

**Figure 2:**
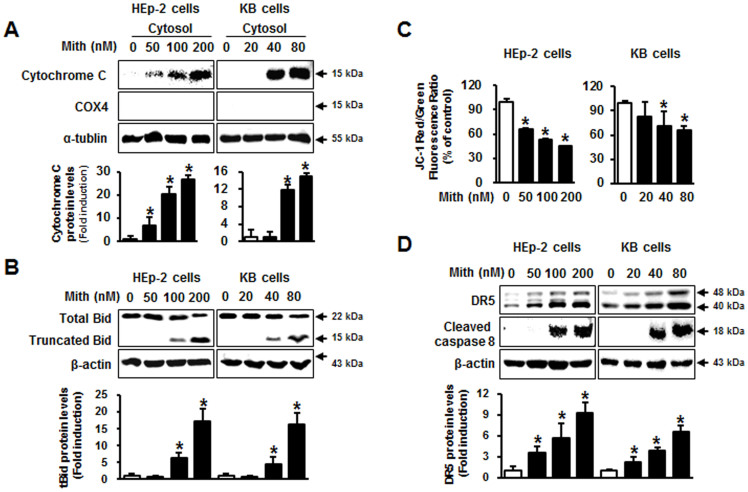
Effect of Mith on a mitochondria-mediated apoptotic pathway. HEp-2 and KB cells were treated with or without Mith for 48 h. (A) Cytochrome c release in Mith-induced apoptosis was evaluated by analyzing cytosolic fractions for cytochrome c by western blot with α-tubulin as internal control for cytosolic fraction and COX4 exclusive mitochondria marker as control for separation of mitochondrial and cytosolic fractions. (B) Western blot of HEp-2 and KB cells for total and truncated Bid. (C) Alteration of ΔΨm after exposure of HEp-2 and KB cells to Mith at indicated concentrations, measured with JC-1 probe as average ratio of red:green fluorescence. (D) DR5 and cleaved caspase-8 by western blot with β-actin as internal control. Results are mean ± SD from three independent experiments. * P < 0.05.

The above results implicated caspase 9-dependent mitochondrial apoptotic pathways. Therefore, we investigated the distribution of cytochrome c in response to Mith treatment. Cytochrome c was released from mitochondria into the cytosol by Mith in a concentration-dependent manner (Fig. 2A). In apoptotic signaling, the BH3-only protein Bid triggers Bak or Bax dimerization, leading to cytochrome c release^21^. In both cervical cancer cell lines, Mith induced Bid truncation at 48 h of treatment (Fig. 2B). We further examined if other anti-apoptotic or pro-apoptotic proteins in the Bcl-2 family responded to Mith in cervical cancer cells. Levels of the pro-apoptotic proteins Bak and Bad significantly increased in KB cells as compared to Hep2 cells (Suppl. Fig. 1). Because a postulated mechanism by which activation of Bid causes cytochrome c release involves changes in ΔΨm, its status in cervical cancer cells before and after Mith treatment was investigated. HEp-2 and KB cells were incubated with JC-1, a fluorescent probe used to measure ΔΨm variations. Mith-treated cells showed a concentration-dependent, rapid, strong decrease in fluorescence intensity (Fig. 2C). Bid is a downstream target of activated caspase-8 death mediated by DR4 or DR5 receptors in certain cancers^22^. Therefore, we investigated whether the levels of DR4, DR5 and cleaved caspase-8 were modulated by Mith treatment. Mith treatment significantly increased the cleaved caspase-8 and DR5 levels compared with negative controls (Fig. 2D). Taken together, these results indicated that Mith induced mitochondria-mediated apoptosis through a DR5/caspase-8/Bid signaling pathway in cervical cancer cells.

### Mith induces proteasome-dependent Sp1 degradation

**Figure 3:**
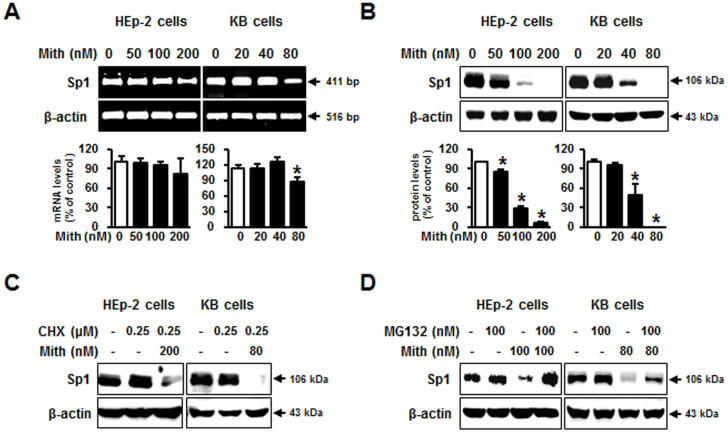
Effect of Mith on Sp1 protein turnover in HEp-2 and KB cells. (A, B) HEp-2 and KB cells were treated with Mith for 48 h. Sp1 mRNA was detected by RT-PCR and protein by western blot. Relative Sp1 levels were quantitated by densitometry and normalized to β-actin. (C) Lysates were from cells pretreated with protein synthesis inhibitor cycloheximide (0.25 μM) for 1 h, then Mith for 48 h. Sp1 was analyzed by western blot. (D) HEp-2 and KB cells were preincubated for 1 h with proteasome inhibitor MG132 (100 nM) before Mith treatment. Sp1 was analyzed by western blot with β-actin was internal control. Results are mean ± SD from three independent experiments. * P < 0.05.

Previous studies suggest that Mith inhibits growth of human cancers by decreasing Sp1 protein^23^,^24^,^25^, but how Sp1 protein is decreased by Mith is not clear. We checked Sp1 mRNA levels in cervical cancer cell lines and saw no significant difference in cells with or without Mith treatment (Fig. 3A). Therefore, we determined if Mith regulated Sp1 protein levels. Mith treatment significantly reduced Sp1 protein in HEp-2 and KB cells (Fig. 3B). To further assess whether Mith affected initiation of Sp1 protein synthesis, HEp-2 and KB cells were incubated with the protein synthesis inhibitor cycloheximide (CHX) before treatment with Mith. CHX treatment did not result in any significant differences in cellular Sp1 levels (Fig. 3C). This result suggested that Sp1 turnover might be related to increased protease activity. Therefore, to determine if decreased levels of Sp1 could be rescued by protease inhibitors, HEp-2 and KB cells were pretreated with the proteasome inhibitor MG132 before Mith treatment. MG132 rescued Sp1 from Mith-induced protein degradation (Fig. 3D), indicating that Mith induced proteasome-dependent Sp1 degradation.

### Mith synergized with Sp1 knockdown to induce apoptosis

Previous studies have suggested that Mith inhibits the growth of various human cancers by decreasing Sp1 protein level^23^,^24^,^25^, but it is still not clear how Sp1 protein level is decreased by Mith. We first checked the mRNA expression levels of Sp1 in different cervical cancer cell lines. No significant difference in the Sp1 mRNA levels was observed in cervical cancer cells with or without Mith treatment (Fig. 3A). Therefore, we next determined whether Mith could regulate Sp1 protein levels. Mith treatment significantly reduced Sp1 protein levels in HEp-2 and KB cell (Fig. 3B). To further assess whether Mith had an effect on the initiation of Sp1 protein synthesis, HEp-2 and KB cells were incubated with the protein synthesis inhibitor cycloheximide (CHX) prior to treatment with Mith. CHX treatment did not result in any significant difference in cellular Sp1 levels (Fig. 3C). The independence of Sp1 degradation upon protein synthesis suggested that Sp1 turnover may be related to increased protease activity. Therefore, to determine whether the decreased levels of Sp1 could be rescued by protease inhibitors, HEp-2 and KB cells were pretreated with the proteasome inhibitor MG132 prior to treatment with Mith. MG132 rescued Sp1 from Mith-induced protein degradation (Fig. 3D), indicating that Mith induces Sp1 degradation in a proteasome-dependent manner.

### Mith synergized with Sp1 knockdown to induce apoptosis

**Figure 4:**
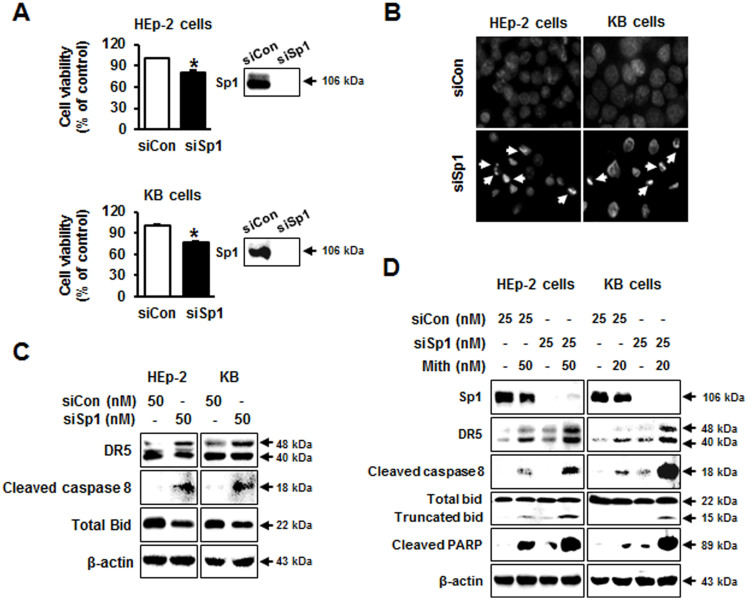
Knockdown of transcription factor Sp1 is synergistic with Mith apoptotic effects. Cells were transiently transfected with control siRNA (siCon) or Sp1-specific siRNA specific (siSp1) for 48 h. (A) Cell viability was determined using MTS assays. (B) By fluorescence microscopy (magnification X400), HEp-2 and KB cells exhibited nuclear fragmentation and condensation after DAPI staining. (C) Bid, DR5, and cleaved caspase-8 were detected by western blot. (D) HEp-2 and KB cells were transiently transfected with Sp1 siRNA for 48 h before Mith treatment. Sp1, DR5, Bid, cleaved caspase-8, cleaved PARP were analyzed by western blot with β-actin as internal control. Results are mean ± SD from three independent experiments. *P < 0.05.

In a Mith-induced apoptosis model, reduced Sp1 protein levels were observed earlier than other mitochondria-mediated apoptosis regulators, specifically DR5, caspase-8, caspase-3 and PARP (Suppl. Fig. 2). We therefore hypothesized that Sp1 might act as a direct upstream regulator of the DR5/caspase-8/Bid signaling pathway. To address more specifically the direct regulatory roles of Sp1 in Mith-mediated DR5/caspase-8/Bid signaling pathway, endogenous Sp1 was knocked down in HEp-2 and KB cells by siRNA. Western blots confirmed reduction of Sp1 protein levels following transfection of cells with Sp1 siRNA (Fig. 4A). Reduction of Sp1 protein was significantly associated with suppression of cell viability (Fig. 4A) and elevated rates of apoptosis (Fig. 4B). Sp1 knockdown also significantly increased the level of cleaved caspase-8, DR5, and truncated Bid compared with that of the negative controls (Fig. 4C). We also determined if the effect of Sp1 down-regulation on cervical cancer cell apoptosis was sensitized by Mith treatment. HEp-2 and KB cells transfected with Sp1 siRNA were exposed to 20 or 50 nM of Mith. The combination of Mith treatment and Sp1 knockdown significantly increased HEp-2 and KB cell t (Fig. 4D).

### Therapeutic effects of Mith in xenograft mouse models of human cervical cancer

**Figure 5:**
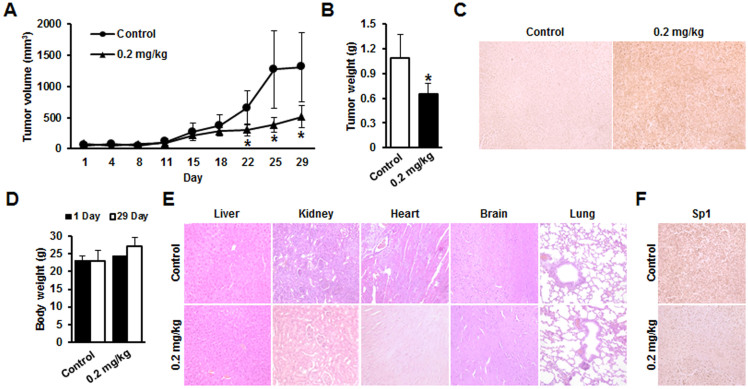
Inhibition of tumor growth by Mith in nude mice xenografted with KB cells. Mice were treated with 0.2 mg/kg/day Mith (i.p.) three times per week for 29 days. (A, B) Treatment with 0.2 mg/kg/day of Mith significantly reduced average tumor volume and weight compared to PBS controls. (C) Representative sections from tumor xenografts from treated mice stained for TUNEL showing cells with fragmented DNA after Mith treatment and vehicle-treated controls. (D, E) Body weight and microscopic pathology of major organs with no evidence of adverse systemic toxicity up to 0.2 mg/kg/day Mith in nude mice xenografted with KB cells. (F) Sp1 immunohistochemical staining of KB cell tumor xenografts. Number of animals per group = 10. Results are mean ± SD from three independent experiments. * P < 0.05.

We further determined the antitumorigenic activity of Mith (0.2 mg/kg/day) in a xenograft model and observed reduction in tumor volume and weight (Fig. 5A and 5B). No significant mouse body weight loss was observed in Mith-treatment groups, indicating that Mith-associated toxicity was minimal (Fig. 5D). Mith also increased TUNEL-positive cells in tumor xenografts (Fig. 5C). No notable intergroup differences were observed among organs, indicating no marked signs of systemic toxicity at the Mith dose used in this study (Fig. 5E). We observed significant inhibition of Sp1 expression in tumor xenografts from Mith-treated mice compared with control mice (Fig. 5F).

## Discussion

Nearly half a million new cervical cancer cases occur among women per year and cervical cancer is responsible for 274,000 deaths. Cervical cancer is the third most common cancer among women and the third leading cause of death^26^. The standard treatment for cervical cancer is cisplatin-based chemotherapy after surgery, which usually yields positive results in initial treatment^27^. In many cases, however, chemoresistance is induced by prolonged cisplatin-based treatment, which has significant toxicity^28^. Although the effects of other chemotherapy drugs including ifosfamide, paclitaxel, and topotecan have been assessed as single drugs or in a combination of drugs, chemotherapy results remain unsatisfactory^18^. Therefore, innovations in cervical cancer treatment mainly aim for more therapies that are effective and better tolerated than existing choices. Sp1-targeted drug is an attractive option for cervical cancer treatment.

Mith is a FDA-approved chemotherapeutic agent that is effective against testicular carcinoma and chronic myeloid leukemia. In a clinical investigation of 305 patients with testicular tumors treated with Mith, 10.8% of patients (33 patients) showed a complete disappearance of tumor in testis and an additional 26.2% of patients (80 patients) was responded with significant regression of tumor in multiple site^10^. In other clinical trials of 13 patients with accelerated phase of chronic myeloid leukemia, 2 patients had been stabilized, 3 patients had partial responses, and 1 patient had a complete response with a major cytogenic response^11^. Recently, our group reported the antitumor effects Mith on human prostate^29^ and oral^30^ cancer cells *in vitro* and *in vivo*. In addition, recent studies suggest that Mith sensitizes cancer cells to tumor necrosis factor (TNF)-induced apoptosis and suppresses p53-mediated transcriptional responses^31^,^32^. Mith, in part, binds selectively to GC-rich sequences in the DNA minor groove and preferentially blocks binding of transcription factors such as Sp1 to guanosine-cytosine (G-C)-rich elements in target gene promoters^33^. Sp1 regulates several cellular functions and influences tumor growth by controlling expression of genes related to tumor growth and development such as cyclin D1, c-Jun, and c-Myc^34^. Previous studies indicate that Sp1 accumulates in tumors cervical tissue^35^. Both stability and transcriptional activity contribute to enhanced Sp1 protein levels observed during tumorigenesis^35^. Previous studies suggest that Mith inhibits the growth of various human cancers by decreasing Sp1 protein^23^,^24^,^25^, but how Sp1 protein is decreased by Mith is unclear. Many transcription factors can be degraded by the proteasome^36^. In many cases, domains of transcriptional activation and degradation signals directly overlap. However, regulation of Sp1 protein levels via proteasome-dependent degradation as not been studied as a mechanism for controlling the amount of Sp1 in cancer cells. Here, we show that Mith decreased Sp1 protein levels by inducing proteasome-dependent degradation. The proteosome inhibitor MG132 rescued Sp1 from Mith-induced protein degradation, indicating that Mith induced degradation via proteasome.

In this study, we investigated the antitumorigenic activity of Mith in cervical cancer cell lines and cervical tumor xenografts. Systemic toxicity that causes acute and chronic organ damage is a major limitation to the clinical application of Mith. Therefore, we assessed the doses at which Mith could be safely administered to mice. Evaluation of multiple organ toxicity revealed that prolonged Mith treatment (0.2 mg/kg) was well tolerated with minimal reduction of body weight upon systemic administration to cervical cancer-bearing mice. Along with recent studies on breast^37^, prostate^29^, and ovarian cancers^38^, our results suggest that Mith is be a good therapeutic candidate for treatment of cancers in which Sp1 is important for promoting and developing disease.

Recent data suggest that induction of tumor cell death by stimulating mitochondrial and death receptor-mediated apoptotic pathways might contribute more to chemoresistance than previously thought^31^,^39^. Therefore, strategies to increase death receptor (DR5)-mediated apoptosis, for example by Mith, might be useful for treatment of chemoresistant cancers. In this study, the apoptotic response to Mith was accompanied by activation of a mitochondria-mediated apoptotic pathway through DR5/caspase-8 signaling. This phenomenon might also explain in part how Mith increases cell sensitivity to TNF-α^31^, as caspase-8 is implicated in TNF-mediated and death receptor-mediated apoptotic pathways^40^. Several regulators and effectors controlling the DR5-mediated apoptotic pathway might be involved in Mith-induced apoptosis. Our hypothesis for Mith function in mediating cervical cancer cell apoptosis is modulation of Bid expression. Truncated (t)Bid, a BH3-only protein of the Bcl-2 family, triggers rapid and extensive release of cytochrome c and subsequent downregulation of apoptosome formation^41^. We observed that in both tested cervical cancer cell lines, Mith induced Bid truncation at 48 h after treatment. Further characterize of truncated (t)Bid to understand its role in Mith-induced apoptosis will be interesting for our future studies. Since Mith has been used clinically for many years to treat various cancers, the inhibitory effects of Mith on cervical cancer are intriguing and merit further study to realize the full potential of Mith in death receptor-mediated apoptosis as an novel therapeutic approach.

In conclusion, we demonstrated that Mith significantly inhibited cervical cancer growth *in vitro* and *in vivo*. At the molecular level, Mith dramatically induced proteasome-dependent Sp1 degradation, thereby suppressing growth of cervical cancer cells through a DR5/caspase-8/Bid signaling pathway. Collectively, these data provide further evidence of the relevance of Sp1 as a therapeutic target and the potential of Mith as an effective therapeutic strategy for cervical cancer.

## Author Contributions

E.S.C. and J.S.N. designed and performed experiments, analyzed data and wrote manuscript draft. J.Y.J. designed and performed experiments and N.P.C. and S.D.C. designed the research plan, analyzed data and finalized manuscript. All authors reviewed the manuscript.

## Supplementary Material

Supplementary Information Supplementary information
