# Supplementary material for: Modulation of specificity protein 1 by mithramycin A as a novel therapeutic strategy for cervical cancer
Source: Sci Rep. 2014 Nov 24;4:7162. doi: 10.1038/srep07162 (PMC4241519; doi:10.1038/srep07162)
Supplement: Supplementary Information [file srep07162-s1.doc]

**Modulation of specificity protein 1 by mithramycin A**

**as a novel therapeutic strategy for cervical cancer**

Eun-Sun Choi, Jeong-Seok Nam, Ji-Youn Jung, Nam-Pyo Cho, Sung-Dae Cho

**Supplementary Figure 1.**

Supplementary Figure 1. The effect of Mith on Bcl-2 family members. HEp-2 and KB cells were treated with Mith, as indicated, for 48 hr, whole cell protein lysates were prepared, and Bcl-2 family members (Bax, Bak, Bad, Bcl-2, Bcl-xL, Mcl-1) were detected by western blot analysis.

**Modulation of specificity protein 1 by mithramycin A**

**as a novel therapeutic strategy for cervical cancer**

Eun-Sun Choi, Jeong-Seok Nam, Ji-Youn Jung, Nam-Pyo Cho, Sung-Dae Cho

**Supplementary Figure 2.**

Supplementary Figure 2. A time-course effect of Mith on apoptosis and Sp1-related signaling pathway. HEp-2 and KB cells treated with Mith for 6, 12, 24 and 48 h, and Sp1, DR5, cleaved caspase-8, Bid, cleaved caspase-3 and PARP were detected by western blot analysis.
